# Editorial: Mechanisms of metastasis from circulating and disseminated tumor cells

**DOI:** 10.3389/fcell.2024.1386050

**Published:** 2024-02-27

**Authors:** Roberto Piñeiro

**Affiliations:** ^1^ Translational Medical Oncology Group (Oncomet), Health Research Institute of Santiago de Compostela, Santiago de Compostela, Spain; ^2^ European Liquid Biopsy Society (ELBS), Hamburg, Germany

**Keywords:** metastasis, circulating tumor cells (CTCs), disseminated tumor cells (DTCs), tumor microenvironment (TME), cancer-associated fibroblasts (CAFs), liquid biopsy

This Research Topic explores the molecular and cellular mechanism underlying the metastatic cascade. The success of cancer cells along metastasis is conditioned by cancer cell intrinsic and extrinsic mechanisms and factors, such as the acquisition of metastasis-facilitating traits, survival to immune attack and mechanical stresses at the tumor site and while in circulation as circulating tumor cells (CTCs), and adaptation to the target organ as disseminated tumor cells (DTCs), which are closely related and depend on the interaction with the components of the tumor microenvironment (TME). Understanding all these processes will pave the way for the identification of new cancer biomarkers and the design of more efficient therapeutic strategies.

CTC survival depends on several factors, including the ability to evade the immune system, resistance to apoptosis/anoikis, and resistance to shear forces and mechanical stress (Wang et al., 2018). Cancer stem cells, a subset of cells thought to play a role in tumor initiation, progression, recurrence, and resistance to therapies (Ayob and Ramasamy, 2018; Li et al., 2023), may be the precursors of CTCs with stem-like phenotype and enhanced metastatic competence (Luo et al., 2018; Ring et al., 2023). Quartieri et al. evidenced that exposure of osteosarcoma cells to hypoxia and radiation increased the population of cells with stem-like properties, with a more aggressive cell phenotype, determined by upregulation of CD133 and TrkB markers and enhanced resistance to anoikis, sphere formation, and sphere clustering capacity. Moreover, chronic hypoxia increases CD47 expression, a signal that helps CTCs evade the immune system.

In relation to mechanical stress, Kurma and Alix-Panabières focused their review on the mechanobiology underlying the survival strategies and adaptations of CTCs in the microcirculation, highlighting the role of fluid shear stress and other mechanical cues on the nuclear envelope and cytoskeleton of CTCs, as well as on the signaling pathways and molecular players activated. It remains crucial to elucidate the involvement of fluid mechanics in the metastatic cascade to design anti-metastasis therapeutic strategies, as well as improve the detection of CTCs by liquid biopsy tests. Even more so in tumors such as breast cancer (BC), a disease in which the spread of cancer cells is an early event that can go unnoticed, and about 20% of patients will have a distant recurrence in the years following adjuvant therapy (Pan et al., 2017). Targeting minimal residual disease, in the form of DTCs, represents a window for therapeutic intervention, but it requires a better understanding of the timing of tumor cell dissemination and the cell-intrinsic and extrinsic factors that drive tumor cell growth. Ring et al. presented a comprehensive review of the knowledge on these key aspects in BC. They discussed the main therapeutic strategies proposed to target dormant cells and highlighted potential areas of improvement for curative cancer care, such as the development of better diagnostic tests - including the potential of liquid biopsy tests and biomarkers associated - and the design of clinical trials.

Tumor-stroma interactions play a significant role in tumor development and metastasis. In recent years, the role of cancer-associated fibroblasts (CAFs) in cancer progression, the largest population of cells in the TME, has received great attention. Previous studies have shown the biological implications of CAFs in CTC survival and proliferation, and the clinical implications of circulating CAFs and heterotypic CTC clusters (Duda et al., 2010; Ao et al., 2015; Ortiz-Otero et al., 2020). In this context, Hurtado et al., studied the interactions between CTCs and CAFs within heterotypic clusters during circulatory dissemination in the zebrafish metastasis model. The clustering of CTCs and CAFs allows the establishment of cell-cell interactions that favor the survival and proliferation of disseminated BC CTCs. These results led the authors to speculate that CTC-CAF interaction in clusters may select a subpopulation of BC CTCs with a higher capacity to survive and proliferate.

In pancreatic ductal adenocarcinoma (PDAC), CAFs and extracellular matrix proteins are involved in the desmoplastic stromal reaction, a key determinant of aggressiveness and poor prognosis. PDAC lacks prognostic and predictive biomarkers that could guide clinical decisions (Sturm et al., 2022). In this regard, as highlighted by Götze et al., circulating CAFs (cCAFs) could be potential new liquid biopsy biomarkers, as cCAFs play a significant role in facilitating CTC-mediated metastasis. The authors shared some insights into the combined detection of CTCs, CAFs and stroma-derived proteomic signatures as prognostic markers, and discussed the targeting of CAFs as potential anti-stromal therapeutic strategies for PDAC. Deepening into the contribution of CAFs to tumor progression, Sari et al., reviewed the impact that autophagy has on their biology. Autophagy, a catabolic process considered a protective mechanism against stress conditions that suppresses tumor growth (Panda et al., 2015), is closely related to cancer pathogenesis (Debnath et al., 2023). Autophagy seems to be a key mechanism for fibroblast activation and CAF formation, triggered by stresses in the TME such as oxidative stress and hypoxia. It also plays a role in the metabolic reprogramming of CAFs, supporting CAF survival and cancer cell metabolism, and activation of autophagy in CAFs contributes to treatment resistance and metastasis. Therefore, a better understanding of autophagy in the TME may be important for the identification of potential therapeutic opportunities.

The dynamic intercellular communication between cancer cells and cells from the TME plays a significant role in metastasis. Cancer cells release abundant extracellular vesicles (EVs), membranous nano-structures playing a role in intercellular communication through the transport and exchange of their cargo (Kalluri and McAndrews, 2023). The review by Dong et al. discussed the role of EVs concerning triple-negative BC metastatic niche establishment, immunosuppression, and enhanced resistance to therapies. EVs hold the potential to become tools for early detection, prognosis evaluation, and recurrence monitoring, guiding clinical decisions, but also as potential carriers of anti-tumor agents. However, some critical aspects in EV research must first be addressed, such as the standardization of EV isolation and storage, optimization of methodologies for profiling EVs’ cargo, and a better molecular understanding of the crosstalk between the EVs from different cell populations in the TME.

In conclusion, the articles included in this Research Topic provide a clear picture of the value that a thorough understanding of the biology of the cells responsible for metastasis and their interaction with stromal cells (i.e., CAFs) can have for the identification of new biomarkers of the metastatic process and the development of new therapeutic strategies. We believe that directing our research efforts in this direction will have a significant impact on how we manage cancer patients to limit the spread of the disease.
